# Continuous Intrajejunal Levodopa–Carbidopa Infusion in Parkinson's Disease Associated with 22q11.2 Deletion Syndrome: A Case Series

**DOI:** 10.1002/mdc3.70738

**Published:** 2026-07-09

**Authors:** Valle Victor Andrés, Jardel Amory, Hopes Lucie, Clément Guillemette, Lambert Laetitia, Becker Aurélie, Dexheimer Mylène, Bonnet Céline, Frismand Solène, Renaud Mathilde, Puisieux Salomé

**Affiliations:** ^1^ Université de Lorraine, Inserm, NGERE Nancy France; ^2^ Service de génétique clinique CHRU de Nancy Nancy France; ^3^ Laboratoire de Génétique Médicale, CHRU Nancy Nancy France; ^4^ Service de Neurologie CHRU de Nancy Nancy France; ^5^ Service de Neurologie CHR de Mercy Ars‐Lequenexy France

**Keywords:** 22q11.2 deletion syndrome, early‐onset Parkinson's disease, genetic parkinsonism, levodopa–carbidopa intestinal gel, psychiatric comorbidity

## Abstract

**Background:**

22q11.2 deletion syndrome (22q11DS) is a multisystem genetic disorder associated with a significantly increased risk of early‐onset Parkinson's disease (EOPD). Management is challenging because psychiatric and cognitive comorbidities often limit advanced therapies such as deep brain stimulation (DBS).

**Cases:**

We report 2 patients with 22q11DS who developed EOPD at about age 30 with prominent psychiatric manifestations. In both cases, diagnosis of 22q11DS was delayed until genetic testing was performed for atypical parkinsonism associated with intellectual disability and dysmorphic features. Severe motor fluctuations and dyskinesia developed early. Because of psychiatric vulnerability, DBS and dopamine agonists were considered unsuitable. Continuous intrajejunal levodopa–carbidopa infusion (LCIG [levodopa–carbidopa intestinal gel]) was initiated, which led to sustained improvement in motor fluctuations and functional status, although individualized dose adjustments were required.

**Conclusions:**

LCIG may represent a valuable therapeutic option in selected patients with 22q11DS‐associated Parkinson's disease when psychiatric comorbidity limits other advanced therapies.

The 22q11.2 deletion syndrome (22q11DS) is one of the most common recurrent microdeletion disorders and results from a heterozygous deletion on chromosome 22q11.2, typically spanning ~3 Mb.^1^ Its clinical presentation is highly variable and may include congenital heart defects, palatal abnormalities, immune dysfunction, hypocalcemia, characteristic facial features, developmental delay, and a significantly increased risk of psychiatric disorders.^1^ Because of its multisystem nature and phenotypic heterogeneity, the condition is frequently underrecognized or diagnosed late.^1^

Beyond neurodevelopmental and psychiatric manifestations, increasing evidence indicates that 22q11DS is associated with a substantially elevated risk of early‐onset Parkinson's disease (EOPD), making it one of the most important genetic risk factors for parkinsonism outside of monogenic Parkinson's disease (PD).^1^ Management in this population is complex. Although levodopa (l‐dopa) remains the cornerstone of treatment, patients with EOPD often develop early motor fluctuations and dyskinesia. Deep brain stimulation (DBS) may be considered high risk because of frequent psychiatric and cognitive comorbidities. Continuous intrajejunal levodopa–carbidopa infusion (levodopa–carbidopa intestinal gel [LCIG]) provides stable dopaminergic stimulation and represents a potential alternative, but experience in 22q11DS remains limited. We report 2 patients with 22q11DS‐associated EOPD treated with LCIG.

## Case 1

**FIG. 1:**
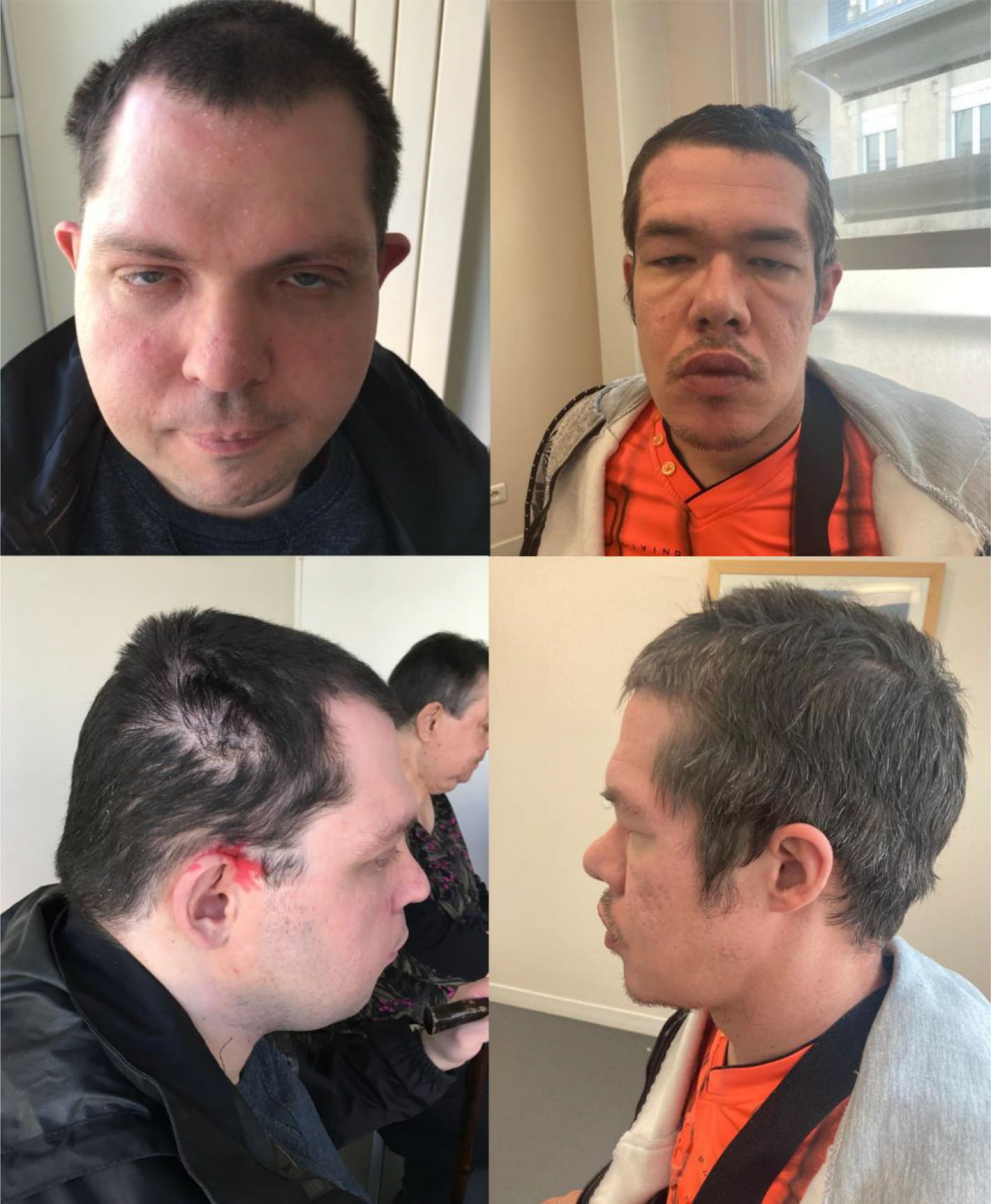
Front and profile photographs of 2 patients with 22q11.2 deletion syndrome, illustrating characteristic dysmorphic features. Patient 1 (left) exhibits amimic face, widow's peak, protruding ears, hooded or puffy upper eyelids, a broad tubular nose with a bulbous tip, low‐set ears, microstomia, and retrognathia. Patient 2 (right) shows a lengthened upper face, hooded or puffy upper eyelids, a broad tubular nose, and malar flattening.

The first patient had mild intellectual disability, facial anomalies (Fig. 1), and delayed psychomotor development. He attended specialized schooling, later obtained vocational training, and entered the workforce. At age 32, he developed a right‐sided resting tremor and was diagnosed with PD, with good dopaminergic responsiveness. At age 34, he developed motor fluctuations. At age 35, during treatment adjustment, he developed hallucinations, delusions, and agitation requiring clozapine.

A Parkinson and movement disorders gene panel detected a heterozygous deletion of the *COMT* gene classified as a variant of uncertain significance. Further evaluation revealed that this finding belonged to a larger chromosomal deletion. Genetic assessment noted facial dysmorphism (Table 1) in association with intellectual disability and EOPD, raising suspicion of 22q11DS. Chromosomal microarray confirmed a heterozygous interstitial deletion of ~2.8 Mb in 22q11.21, consistent with the typical 22q11DS deletion.

**TABLE 1:** Clinical features of 22q11.2 deletion syndrome in the 2 patients

Feature	Typical in 22q11DS	Patient 1	Patient 2
Congenital heart disease	Common	No	No
Palatal abnormalities	Frequent	Not documented	Not documented
Immune dysfunction	Possible	No	No
Learning difficulties/intellectual disability	Very common	Mild intellectual disability	Learning difficulties
Characteristic facial dysmorphism	Common	Yes	Yes
Hearing loss	Possible	No	No
Psychiatric comorbidity	Very common	Psychosis	Schizophrenia
Skeletal anomalies	Possible	No	No
Genitourinary anomalies	Possible	No	No
Hypoparathyroidism/hypocalcemia	Common	Yes	Yes

Abbreviation: 22q11DS, 22q11.2 deletion syndrome.

Approximately 5 years after diagnosis, the patient developed significant motor fluctuations with severe end‐of‐dose wearing‐off episodes (≈50% of the daytime), persistent nocturnal off periods, and disabling peak‐dose l‐dopa‐induced dyskinesias (≈20%), reflecting a narrow therapeutic window (Table 2). The levodopa equivalent daily dose (LEDD) was estimated at ~1800 to 2000 mg/day. In addition, psychiatric symptoms (hallucinations and delusions) were sensitive to dopaminergic dose adjustments, further complicating management.

**TABLE 2:** Neurological characteristics and treatment response in 22q11DS‐associated early‐onset Parkinson's disease

Feature	Patient 1	Patient 2
Age at symptom onset	32	29
Age at motor fluctuations	34	31
Initial presentation	Right resting tremor	Right resting tremor
Rigidity	Bilateral (right > left)	Bilateral (right > left)
Bradykinesia/akinesia	Bilateral (right > left)	Bilateral (right > left)
Camptocormia/truncal dystonia	No	Yes
Freezing of gait	Yes	Yes
Dysarthria	Progressive	Persistent (nasal voice)
Dysautonomia	Mild	Urinary incontinence, erectile dysfunction
Psychiatric manifestations	Delusions, hallucinations, agitation	Hallucinations, delusions, anxiety
Initial levodopa response	Good	Good (challenge test UPDRS III: 79 → 40; **≈**49%)
Antipsychotic treatment	Clozapine	Clozapine
Dopamine transporter imaging	Not performed	Abnormal
Confirmed 22q11.2 deletion	Yes (microarray)	Yes (microarray + FISH)
Age at LCIG initiation	37	37
LEDD before LCIG	1800–2000 mg/day	1800–2000 mg/day
LEDD after LCIG	1300–1400 mg/day during daytime (at 3 mL/h during daytime and 2 mL/h at night, morning bolus of 3 mL)	1800–2000 mg/day (5.5 mL/h in the morning and 6.3 mL/h in the afternoon, morning bolus of 5.5 mL)
Motor fluctuations before LCIG	Severe: severe wearing‐off episodes (≈50% of the daytime); persistent nocturnal OFF periods; disabling peak‐dose dyskinesias (≈20% of the daytime)	Severe: severe and prolonged wearing‐off episodes (inability to walk, frequent falls, and intense pain, ≈40%–60% of the daytime)
Motor fluctuations after LCIG	Wearing‐off resolved; persistent peak‐dose dyskinesias, less disabling and better tolerated	Near‐complete wearing‐off control; residual late‐afternoon wearing‐off episode; mild and nontroublesome peak‐dose dyskinesias
Psychiatric stability on LCIG	Stable	Stable
Outcome	Death at 40 years (PD‐related complications) (3 years of follow‐up)	Ongoing follow‐up (3 years of follow‐up)

Abbreviations: 22q11DS, 22q11.2 deletion syndrome; LCIG, levodopa–carbidopa intestinal gel; LEDD, levodopa equivalent daily dose; PD, Parkinson's disease; UPDRS, Unified Parkinson's Disease Rating Scale.

Due to the combination of severe motor fluctuations with significant l‐dopa‐induced dyskinesias and wearing‐off episodes, and a narrow therapeutic window with dose‐sensitive psychiatric complications, LCIG was considered the most appropriate advanced therapy to provide continuous dopaminergic stimulation while minimizing peak‐dose‐related adverse effects. DBS and apomorphine infusion were not retained due to psychiatric comorbidity and overall clinical complexity.

At age 37, LCIG therapy was initiated via gastrojejunostomy (3 mL/h daytime, 2 mL/h night; morning bolus 3 mL), leading to significant improvement in motor fluctuations, despite a reduction in the LEDD (1300–1400 mg/day). Under LCIG, wearing‐off episodes resolved, whereas peak‐dose l‐dopa‐induced dyskinesias persisted but were less disabling and better tolerated (Table 2).

At age 40, progressive dysphagia led to weight loss and required enteral nutritional support. The patient subsequently died from infectious complications related to advanced dysphagia in the context of PD.

## Case 2

The second patient had, in addition to facial anomalies (Fig. 1; Table 1), childhood learning difficulties and left school early but later completed vocational training. During adolescence, he developed anxiety followed by heavy alcohol and cannabis use. Persistent psychotic symptoms led to a diagnosis of schizophrenia at age 37, treated with clozapine.

At age 29, he developed a right‐sided resting tremor followed by progressive akinesia and rigidity with a clinically significant dopaminergic response. At age 31, he developed motor fluctuations (Table 2). He later developed camptocormia, freezing of gait, and falls. Rehabilitation combined with l‐dopa and pramipexole improved mobility, but wearing‐off periods persisted. Pramipexole was discontinued due to impulse control disorder and replaced with levodopa–carbidopa.

At age 37, the patient presented with severe and prolonged wearing‐off episodes characterized by an inability to walk, frequent falls, and intense pain, which tended to occur randomly and accounted for up to 40% to 60% of the daytime (Table 2). Unfortunately, although increasing the intensity of l‐dopa medication helped reduce wearing‐off episodes, it also led to significant psychiatric side effects, including delirium and hallucinations, which considerably limited the scope for therapeutic intervention. Motor worsening was also accompanied by dysarthria, dysphagia, and dysautonomia. LEDD was estimated at ~1800 to 2000 mg/day. Due to this psychiatric vulnerability and sensitivity, continuing clozapine monotherapy was considered the safest option.

The association of EOPD, intellectual disability, and facial dysmorphism prompted genetic testing. Chromosomal microarray and confirmatory FISH (Fluorescence in situ hybridization) demonstrated a heterozygous 22q11.21 deletion consistent with 22q11DS. Cardiac evaluation was normal.

In this context, and similar to case 1, LCIG was selected as the most appropriate therapeutic option to ensure continuous dopaminergic stimulation, reduce motor fluctuations, and minimize neuropsychiatric adverse effects in a patient with a highly fragile psychiatric profile.

LCIG therapy was initiated with rapid improvement in motor fluctuations. The patient had sustained benefit, with near‐complete wearing‐off control and only mild, nontroublesome peak‐dose dyskinesias. A residual late‐afternoon wearing‐off episode remains but consistently responds to a 4‐mL bolus. After a temporary interruption due to infection, LCIG was restarted with individualized dosing (5.5 mL/h in the morning and 6.3 mL/h in the afternoon, with a morning bolus of 5.5 mL; estimated LEDD ~1800–2000 mg/day) with sustained motor improvement and no further psychiatric decompensation over >3 years of follow‐up.

## Discussion

This case series highlights the association between EOPD and 22q11DS and illustrates the therapeutic challenges related to psychiatric and cognitive comorbidities.^1^ In both patients, EOPD developed at about age 30 and was accompanied by early psychiatric manifestations, a combination strongly suggestive of underlying 22q11DS.^2^ Diagnosis is frequently delayed because of phenotypic heterogeneity and incomplete expression of classical features.^2^ Psychiatric symptoms often precede neurological manifestations and may obscure the diagnosis of PD, particularly in patients exposed to antipsychotic medications.^3^ Dopamine transporter imaging can be valuable in differentiating neurodegenerative parkinsonism from drug‐induced parkinsonism in such contexts.^4^

The mechanisms linking 22q11DS and PD remain incompletely understood. Haploinsufficiency of multiple genes within the deleted region, combined with an increased burden of rare variants affecting dopaminergic pathways, may contribute to neurodegeneration.^5^ Although *COMT* deletion has not been definitively associated with PD, dopaminergic dysregulation and oxidative stress have been proposed as potential mechanisms.^6^

Importantly, contemporary bioinformatics approaches allow detection not only of point mutations but also of chromosomal rearrangements, including 22q11.2 deletions. In one of our patients, the deletion was initially flagged through copy number variation analysis included in a Parkinson and movement disorders gene panel, underscoring the usefulness of comprehensive genetic assays. This observation raises the possibility that genome‐wide testing may represent a more efficient first‐line diagnostic approach in EOPD, enabling simultaneous evaluation for PD‐related genes and structural chromosomal abnormalities.

Management of PD in 22q11DS is particularly complex. As in other forms of EOPD, motor fluctuations and dyskinesia occur early and may be severe. Psychiatric and cognitive comorbidities frequently limit advanced therapies. DBS may worsen psychiatric symptoms, and dopamine agonists may exacerbate psychosis or impulse control disorders.^7^ In this context, LCIG provides continuous dopaminergic stimulation and may reduce motor fluctuations without destabilizing psychiatric status.

In both patients, LCIG produced sustained improvement in motor fluctuations and functional status, although individualized dose adjustments were required to manage dyskinesia. These findings support the feasibility of this therapy in carefully selected patients with 22q11DS and severe psychiatric comorbidity. Emerging continuous subcutaneous l‐dopa formulations may provide less‐invasive alternatives in the future.^8^

From a practical standpoint, our observations suggest that LCIG should be considered a preferred advanced therapy in patients with 22q11DS‐associated PD when psychiatric vulnerability limits the use of DBS or dopamine agonists. Careful psychiatric stabilization, individualized dose titration, and multidisciplinary follow‐up are essential to optimize outcomes. More broadly, clinicians should consider systematic genetic evaluation in patients presenting with EOPD associated with psychiatric symptoms, intellectual disability, or dysmorphic features. Early identification of 22q11DS may reduce diagnostic delay, guide therapeutic decisions, and improve multidisciplinary management, including genetic counseling and surveillance for systemic complications.

## Author Roles

(1) Research project: A. Conception B. Organization C. Execution; (2) Manuscript preparation: A. Writing of the first draft. B. Review and critique.

S.P.: 1A, 1B, 1C, 2A, 2B

V.A.V.: 1B, 1C, 2A

A.J.: 1A, 1B

L.H.: 1A

C.G.: 1A

L.L.: 1A

A.B.: 1A

M.D.: 1A

C.B.: 1A

S.F.: 1A

M.R.: 1A, 1B, 2B

## Disclosures

**Ethical Compliance Statement:** This study was approved by the Institutional Review Board of Nancy University Hospital. Written informed consent for publication of clinical data and identifiable images was obtained from the patient (or their legal representative) and documented in the medical record. We confirm that we have read the journal's position on issues involved in ethical publication and affirm that this work is consistent with those guidelines.

**Funding Sources and Conflicts of Interest:** All authors have completed the ICMJE disclosure form and declare no conflicts of interest related to this work.

## Financial Disclosures and Conflicts of Interest

Author disclosures are available in the Supporting Information.

## Supporting information


**Data S1.** COI_Disclosure.

## Data Availability

The data that support the findings of this study are available on request from the corresponding author. The data are not publicly available due to privacy or ethical restrictions.
